# Neuromonitoring Correlates of Expertise Level in Surgical Performers: A Systematic Review

**DOI:** 10.3389/fnhum.2022.705238

**Published:** 2022-02-16

**Authors:** Theodore C. Hannah, Daniel Turner, Rebecca Kellner, Joshua Bederson, David Putrino, Christopher P. Kellner

**Affiliations:** ^1^Department of Neurosurgery, Icahn School of Medicine at Mount Sinai, New York, NY, United States; ^2^Silicea Labs, Los Angeles, CA, United States; ^3^Department of Rehabilitation Medicine, Icahn School of Medicine at Mount Sinai, New York, NY, United States

**Keywords:** neural mechanism, surgical expertise, choking effect, distraction, temporal demand

## Abstract

Surgical expertise does not have a clear definition and is often culturally associated with power, authority, prestige, and case number rather than more objective proxies of excellence. Multiple models of expertise progression have been proposed including the Dreyfus model, however, they all currently require subjective evaluation of skill. Recently, efforts have been made to improve the ways in which surgical excellence is measured and expertise is defined using artificial intelligence, video recordings, and accelerometers. However, these aforementioned methods of assessment are still subjective or indirect proxies of expertise, thus uncovering the neural mechanisms that differentiate expert surgeons from trainees may enhance the objectivity of surgical expertise validation. In fact, some researchers have already suggested that their neural imaging-based expertise classification methods outperform currently used methods of surgical skill certification such as the Fundamentals of Laparoscopic Surgery (FLS) scores. Such imaging biomarkers would not only help better identify the highest performing surgeons, but could also improve residency programs by providing more objective, evidence-based feedback and developmental milestones for those in training and perhaps act as a marker of surgical potential in medical students. Despite the potential advantages of using neural imaging in the assessment of surgical expertise, this field of research remains in its infancy. This systematic review identifies studies that have applied neuromonitoring in assessing surgical skill across levels of expertise. The goals of this review are to identify (1) the strongest neural indicators of surgical expertise, (2) the limitations of the current literature on this subject, (3) the most sensible future directions for further study. We found substantial evidence that surgical expertise can be delineated by differential activation and connectivity in the prefrontal cortex (PFC) across multiple task and neuroimaging modalities. Specifically, novices tend to have greater PFC activation than experts under standard conditions in bimanual and decision-making tasks. However, under high temporal demand tasks, experts had increased PFC activation whereas novices had decreased PFC activation. Common limitations uncovered in this review were that task difficulty was often insufficient to delineate between residents and attending. Moreover, attending level involvement was also low in multiple studies which may also have contributed to this issue. Most studies did not analyze the ability of their neuromonitoring findings to accurately classify subjects by level of expertise. Finally, the predominance of fNIRS as the neuromonitoring modality limits our ability to uncover the neural correlates of surgical expertise in non-cortical brain regions. Future studies should first strive to address these limitations. In the longer term, longitudinal within-subjects design over the course of a residency or even a career will also advance the field. Although logistically arduous, such studies would likely be most beneficial in demonstrating effects of increasing surgical expertise on regional brain activation and inter-region connectivity.

## Introduction

Surgical expertise does not have a clear definition and is often culturally associated with power, authority, prestige, and case number rather than more objective proxies of excellence (Alderson, 2010; Fahy et al., 2019). Multiple models of expertise progression have been proposed, however, they all require subjective evaluation of skill (Howell, 1986; Dreyfus et al., 1997; Hoffman, 1998). Recently, efforts have been made to improve the ways in which surgical excellence is measured and expertise is defined using artificial intelligence, video recordings, and accelerometers (Sánchez et al., 2014; Wang and Majewicz Fey, 2018; Fahy et al., 2019). However, these aforementioned methods of assessment are still subjective or indirect proxies of expertise, thus uncovering the neural mechanisms that differentiate expert surgeons from trainees may enhance the objectivity of surgical expertise validation. Such biomarkers would not only help better identify the highest performing surgeons, but could also improve residency programs by providing more objective, evidence-based feedback and developmental milestones for those in training and perhaps act as a marker of surgical potential in medical students. In fact, one study already suggests that neuromonitoring of a surgical task is a better classifier of expertise than currently used surgical skill certification methods such as Fundamentals of Laparoscopic Surgery (FLS) performance (Nemani et al., 2018).

**FIGURE 1:**
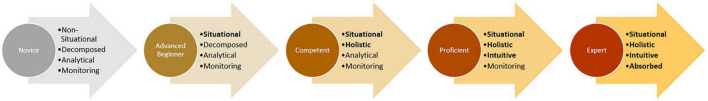
The Dreyfus model of skill acquisition. Skill levels are labeled in the circles and associated states of the four cognitive parameters are written in the arrows. Cognitive parameters in which subjects have achieved the more advanced state are highlighted with bold text.

A commonly used framework of expertise progression is the Dreyfus model for skill acquisition. It describes the progression from novice to expert based on four binary cognitive parameters: Recollection (non-situational or situational), Recognition (decomposed or holistic), Decision (analytical or intuitive), and Awareness (monitoring or absorbed) (Dreyfus and Dreyfus, 1980). Learners go through five stages based on these parameters: Novice, Advanced Beginner, Competent, Proficient, and Expert (Figure 1). The transition from Novice to Advanced Beginner occurs as the subject gains enough experience to develop the ability to use some situational cues rather than non-situational rules to make decisions based their prior experiences. An example of this transformation is when a student-driver begins using the sound of the engine rather than simply the speed on the speedometer to determine when to shift gears. The transition from Advanced Beginner to Competent requires the trainee to process the valence of and integrate the multitude of situational cues in their planning and decision making, taking on personal responsibility for the outcome. For example, whereas an Advanced Beginner exiting the highway at high speed will not have a holistic understanding of this scenario and thus may be overly reliant on a single decomposed cue, such as the sound of the engine, to determine their next step, a competent student-driver will generate a more holistic view of the situation by integrating various cues such the current speed of the vehicle, the curvature of the off-ramp, the condition of the road, the presence of other vehicles, etc. in order to determine whether to shift gears, let off the accelerator, or apply the brakes. The transition from Competent to Proficient occurs as decision making becomes more intuitive than analytical. The proficient driver leaving the highway at very high speed intuitively recognizes the need to slow down and makes faster decisions about how best to mitigate the risk of crashing as they do not spend time consciously integrating various cues. The transition from Proficient to Expert occurs as the subject is able to make increasingly refined discriminations and the time between recognizing the situation and performing the appropriate decision becomes negligible. Whereas a Proficient driver exiting the highway at too high a speed recognizes the situation and intuitively knows what to do, an Expert driver may not even consciously notice their speed, but already has their foot on brake applying precisely the proper amount of force.

Becoming an expert surgeon requires years of consistent training and practice. Two theories of learning commonly incorporated into contemporary teaching in the surgical specialties are cognitive load and deliberate practice (Kavic, 2012). Cognitive load theory contends there are two main forms of memory, working memory and long-term memory (Leppink, 2017). Whereas working memory is finite, the capacity of long-term memory is considered infinite and, through repetition, the transformation of working memory into long-term memory is an essential component of developing expertise. At the neuronal level, this transformation is likely achieved through long term potentiation (LTP) which requires repetitive activation with relevant associations (Zhu et al., 2016). Deliberate practice postulates that expertise is acquired through goal-directed, extensive, consistent, and long-term practice, downplaying the importance of inherited traits or innate abilities (Macnamara and Maitra, 1993; Kavic, 2012). Deliberate practice as the mechanism through which to achieve the cognitive load transformation underlies the framework for the length and intensity of residency training.

Although our understanding of the molecular mechanisms of LTP has been steadily increasing, the ways in which deliberate practice and expert training modulate activation and connectivity within the brains of surgeons over the course of a career is less clear (Nicoll, 2017). The unconscious nature of procedural memory, a type of long-term motor memory responsible for storing learned technical tasks, compared to the conscious and effortful nature of working memory provides an alluring framework to explain obvious differences in performance between novice and expert surgeons on various surgical tasks (Kavic, 2012). However, studies investigating elite athletes have demonstrated that these performers have augmented abilities in numerous modalities, including integrating postural context cues, pattern recognition, and incorporating situational probabilities into decision making. Similarly, surgical expertise likely results in many neural connectivity and activity modifications across cognitive, perceptual, and motor domains (Savelsbergh et al., 2005; Willams et al., 2006; Farrow and Reid, 2012; Roca and Williams, 2016).

Cognitive load theory further postulates there are three forms of cognitive load that consume working memory. There is an intrinsic load which is the working memory required to perform the essential aspects of the task, there is the extraneous load which is working memory used on all non-essential aspects of the task, and then there is the germane load which is the working memory used to create and/or implement strategies or schemas to successfully complete the task (Young et al., 2014). The Dreyfus model of skill acquisition suggests that as expertise level increases and decision making becomes more intuitive than analytical, the germane load should decrease resulting in reduced overall cognitive load. As a corollary of decreased germane load, experts should also be better able to handle increases in extraneous load as a result of distractions, secondary tasks, or increased stress. The brain regions frequently associated with acquisition, consolidation and implementation of task-related schemas are various subregions of the prefrontal cortex, the hippocampus, the medial temporal lobe, and the cerebellum (van Kesteren et al., 2012; Koziol et al., 2014; Spalding et al., 2015; Gilboa and Marlatte, 2017; Guo and Yang, 2020). Thus, differences in these regions may be evident due to changes in the various types of cognitive load as surgical performers progress along the Dreyfus model of expertise even when task performance metrics are similar.

Despite this potential advantage of using neural imaging in the assessment of surgical expertise, this field of research remains in its infancy (Modi et al., 2017). This systematic review identifies studies that have applied neuromonitoring in assessing surgical skill across at least two levels of expertise. The goals of this review are to identify (1) the strongest neural indicators of surgical expertise, (2) the limitations of the current literature on this subject, (3) the most sensible future directions for further study.

## Methods

A systematic review of the literature was conducted of articles in Pubmed and PsychInfo as of November 30th 2021. Additional studies were identified through Google searches and from citations in other publications. All published, English language, full text studies that stratified participants into at least two levels of surgical expertise and conducted neuromonitoring during the performance of a surgery-related task were included. Studies that evaluated surgical expertise without neuromonitoring and studies evaluating neuromonitoring outcomes of a single expertise level were excluded. Review articles were also excluded. The search terms were:

[(“surgery” OR “surgical performance” OR “surgical skill” OR “skill evaluation” OR “surgical expertise” OR “motor skill” OR “laparoscopic task” OR “motor learning”) AND (“brain function” OR “cognitive function” OR “neural function” OR “brain activation” OR “neural network” OR “functional connectivity” OR “connectivity”)] AND (“functional neuroimaging” OR “neuromonitoring” OR “functional magnetic resonance imaging” OR “fMRI” OR “functional near infrared spectroscopy” OR “NIRS” OR “fNIRS” OR “diffuse optical tomography” OR “diffuse optical imaging” OR “electroencephalography” OR “EEG” OR “positron emission tomography” OR “PET” OR “deep learning” OR “artificial intelligence”).

## Results

### Overview

**FIGURE 2:**
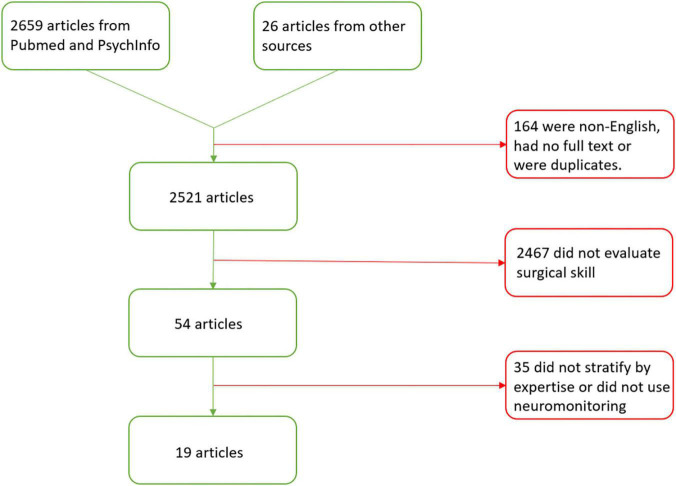
Preferred reporting items for systematic review and meta-analysis (PRISMA) flow diagram for articles evaluating the neural mechanisms of surgical expertise.

A total of 2,659 articles were identified through this method. Another 26 articles were retrieved from other sources (Figure 2). There were 2,521 articles left after titles were excluded for being duplicates, not having a full text, or not being written in English. An additional 2,467 articles were excluded because they did not evaluate surgical skill. Finally, 35 articles were excluded because they were review articles, did not stratify subjects into at least 2 expertise levels or they did not use neuromonitoring. After all exclusions, there were 19 articles included in the review and summarized in Table 1. The studies are presented in sections based on the type of task performed and the brain regions demonstrating significant findings. The task sections are: motor task visualization, motor task observation, motor task performance, distraction, high temporal demand, and decision-making. Brain regions are specified in parentheses for each task section with the exception of the motor task performance section in which brain regions are explored in four subsections.

**TABLE 1:** Summary of the 19 studies identified in the systematic review.

Study (year)	Participants (groupings)	Surgical tasks (paradigm)	Neuromonitoring modality	Brain regions monitored	Task performance results summary	Neuromonitoring results summary
Andreu-Perez et al., 2016	32 participants (9 surgeons, 11 residents, and 12 medical students)	Laparoscopic needle insertion, double-throw knot and single-throw knot (Motor Task Performance)	fNIRS	Functional connectivity between frontal regions (intra-regional) and between frontal and motor regions (inter-regional).	S > R > MS	PFC-SMA, PFC-PMC, SMA-PFC connectivity: ES < MS SMA-M1 connectivity: ES = MS
Duty et al., 2012	10 participants (five surgeons, five medical students)	Watching videos of a peg transfer task and a laparoscopic partial nephrectomy. (Motor Task Observation) Performing peg transfer task. (Motor Task Performance)	PET	Whole brain	ES > MS	**Watching videos** Cerebellum: ES > MS Visual Cortex: ES < MS **Peg transfer task** Insula, precuneus, inferior occipital gyrus: ES < MS
Garbens et al., 2020	18 participants (nine “high performer” medical students and nine “low performer” medical students)	Peg pointing, intracorporeal knot tying, and PicSOr tasks. (Motor Task Performance)	fMRI	Whole brain. The following regions of interest were identified *a priori*: PFC, SMA, PMC, parietal areas, cerebellum, and thalamus	Peg pointing task: not reported Knot tying task speed: HP > LP PicSOr task: HP = LP	SMA: HP < LP
Guru et al., 2015	10 participants [three “expert” surgeons, five “competent/proficient” (C/P) surgeons or fellows, two “beginner” residents]	**Basic tasks:** ball placement, suture pass, peg transfer **Intermediate tasks:** knot tying **Advanced tasks:** simulated urethro-vesical anastomosis. (Motor Task Performance)	EEG	Whole brain	**Basic tasks:** ES = C/P > R **Intermediate tasks:** ES = C/P > R **Advanced Tasks:** ES > C/P	**Basic tasks** Cognitive burden: ES < C/P < R **Intermediate tasks** Cognitive burden: ES < C/P < R **Advanced tasks:** Cognitive burden: ES < C/P
James et al., 2011	29 participants (11 surgeons (>50 endoscopic procedures per year), 18 medical students)	Simulated natural orifice translumenal endoscopic surgery (NOTES) navigation task. (Motor Task Performance)	fNIRS	PFC	ES > MS	LPFC: E > MS (not robust to multivariable regression)
Keles et al., 2021	28 participants (11 surgeons, 17 medical students)	Peg transfer task and Threading task. (Motor Task Performance)	fNIRS	PFC and orbitofrontal cortex	Not reported	Experts had greater PFC activation when self-reported cognitive task load was lower. Whereas novices had greater PFC activation when self-reported cognitive task load was higher. Machine learning methods classified subjects into two expertise categories based on PFC activation with good accuracy.
Kok et al., 2018	30 participants (10 surgeons, 10 residents, 10 medical students)	Observation of video recorded bimanual motor task performed by expert surgeon. (Motor Task Observation)	fMRI	Motor neuron system identified specifically for each individual participant	n/a	Mirror neuron system activation: ES = R = MS
Leff et al., 2006	7 participants (three residents, two novices)	Knot tying. (Motor Task Performance)	fNIRS	Left frontal cortex	Residents > MS	R = MS
Leff et al., 2007	62 participants (19 surgeons, 21 residents, 22 medical students)	Knot tying. (Motor Task Performance)	fNIRS	PFC	ES = R > MS	PFC: ES = R > MS
Leff et al., 2008	62 participants (19 surgeons, 21 residents, 22 medical students)	Knot tying. (Motor Task Performance)	fNIRS	Right parietal cortex and left PFC	ES = R > MS	PFC: ES < MS
Leff et al., 2017	22 participants (5 surgeons, 7 residents, 10 medical students)	Subjects were prompted to make a decision about the next step in a surgical procedure as they watched a video of the procedure. (Decision-Making)	fNIRS	PFC	S = R > MS	Dorsolateral PFC: ES = R < MS
Modi et al., 2018	33 participants (10 PGY-5 residents, 8 PGY3-4 residents, 15 PGY1-2 residents)	Laparoscopic suturing task with time constraints. (High Temporal Demand)	fNIRS	PFC	Knot tensile strength under time constraint: PGY-5 > PGY1-4	PFC activation under time constraint: PGY-5 > PGY1-4
Modi et al., 2019	33 participants (all residents, but stratified by expertise level based on the stability of performance under stress conditions)	Laparoscopic suturing task. (High Temporal Demand)	fNIRS	PFC	n/a	Ventrolateral and dorsolateral PFC activation under time constraints: HP > LP
Morris et al., 2015	9 participants (three surgeons, three residents, three medical students)	Visualization of knot tying by hand. (Motor Task Visualization) Knot tying by hand. (Motor Task Performance)	fMRI	Whole brain	Not reported	Primary visual cortex activation when visualizing task: ES > MS PFC activation when performing task: ES < MS
Mylonas et al., 2012	22 participants (1 expert, 21 novices)	Visuomotor target localization task. (Motor Task Performance)	fNIRS	PFC	Not reported	Frontal lobe activation: ES < N
Nemani et al., 2018	30 participants (8 “Experts”: Senior residents or attending, 9 “Novices”: junior residents, 13 “trainees”: 3 “skilled” medical students, 4 “unskilled” medical students, 5 control medical students (did not undergo training phase)	Pattern cutting task. (Motor Task Performance)	fNIRS	PFC, M1, SMA	Expert surgeons performed better than novice surgeons. After training, trainees performed better than controls.	PFC activation: ES < MS ST < UT Medial M1 and SMA activation: ES > MS ST > UT
Nemani et al., 2021	39 participants (8 surgeons, 9 residents, 22 medical students)	Laparoscopic pattern cutting task in both physical and virtual environments. (Motor Task Performance)	fNIRS with wavelet coherence (WCO) metrics	PFC, M1, SMA	Not reported	CPFC-SMA connectivity: ES < R in physical task ES > R in virtual task Trained MS > Untrained MS
Ohuchida et al., 2009	21 participants (4 surgeons, 4 trainees, 13 novices)	Laparoscopic suturing and knot tying. (Motor Task Performance)	fNIRS	Front parietal cortices	Knot tying: ES > T > N	Frontal lobe activation: ES < T > N
Shetty et al., 2016	35 participants (11 experts, 12 residents, 12 novices)	Laparoscopic suturing drill. (Motor Task Performance)	fNIRS	PFC	Initially: ES > R > N. After 2 weeks of training: ES = N	PFC activation: ES < R < N, initially and after 2 weeks of training.

*C/P, competent/proficient; CPFC, central prefrontal cortex; EEG, electroencephalogram; ES, expert surgeon; fMRI, functional magnetic resonance imaging; fNIRS, functional near-infrared spectroscopy; LPFC, lateral prefrontal cortex; M1, primary motor cortex; MS, medical student; N, novice; NOTES, natural orifice transluminal endoscopic surgery; PET, positron emission tomography; PFC, prefrontal cortex; PGY, post graduate year; PicSOr, pictorial surface orientation; PMC, premotor cortex; R, resident surgeon; SMA, supplementary motor area; ST, skilled trainee; T, trainee; UT, unskilled trainee; WCO, wavelet coherence.*

### Motor Task Visualization (Visual Cortex and Temporal Lobe Activation)

Only one study was identified that evaluated differences in neural activation while visualizing a surgical task. Morris et al. (2015) evaluated three expert surgeons, three residents, and three medical students as they visualized knot tying by hand while undergoing fMRI. The Dreyfus model predicts this type of task to best differentiate the medical students, who have little to no experience tying knots, from the residents and attending as knot tying is a decomposed, basic surgical task that both residents and attending are very familiar. These researchers found that experts had greater activation in the visual cortex and the posterior superior temporal sulcus compared to novices. No comparisons were made between experts and residents. Similar results had previously been noted in expert dancers, however, Morris et al. (2015) was significantly limited by its sample size and has not been corroborated as there were no other studies evaluating surgeons performing visualization tasks (Calvo-Merino et al., 2005). However, there have been two other studies evaluating differences in neural activation across surgical expertise level in motor observation tasks (Duty et al., 2012; Kok et al., 2018).

### Motor Task Observation (Motor Neuron System Activation)

There is a group of interconnected neurons, known as the mirror neuron system (MNS), located in the inferior frontal gyrus, precentral gyrus, inferior parietal lobule, and cortical visual areas that activate in response to observing the actions of others. Importantly, there is differential activation of the MNS depending on whether or not the performed action is part of the observer’s own motor repertoire (Rizzolatti and Craighero, 2004; Rajmohan and Mohandas, 2007). It was hypothesized that, similar to athletes and musicians, activation within the MNS should be modulated by level of expertise in surgeons watching videos of various surgical techniques (Kok et al., 2018). Using positron emission tomography (PET), Duty et al. (2012) demonstrated that whereas medical students had greater blood flow to the visual cortex when watching a laparoscopic nephrectomy for the first time, expert surgeons had increased activation in the posterior cerebellum. Thus, it is critical to differentiate between visualization and observation tasks as Morris et al. (2015) found increased visual cortex activation was positively associated with increasing expertise in visualization tasks, but Duty et al. (2012) found it was inversely associated with expertise in observation tasks. Although the cerebellum is not classically associated with MNS, there is strong evidence that long-term procedural memory involves the cerebellum suggesting this activation pattern is consistent with prior consolidation of a learned motor task (Gabrieli, 1998; Lum et al., 2012). Conversely, using function magnetic resonance imaging (fMRI), Kok et al. (2018) did not find significant differences between novices, intermediates, or expert surgeons in the MNS activation of orthopedic surgeons watching videos of routine orthopedic surgeries. Similar to Morris et al. (2015), the tasks in Duty et al. (2012) and Kok et al. (2018) best differentiate between novice and intermediate/expert surgeons. Consistent with this, there was a marginally significant decrease in MNS activation in intermediates compared to novices, however, this effect was in the opposite direction of what has been observed previously which may indicate that surgical motor representations are encoded differently than athletic or musical motor representations. However, important methodological differences could have played a role in the result of this second study. For example, the sample size was much smaller in Kok et al. (2018) compared to a prior meta-analysis that produced significant results in a cohort of athletes, however another study in dancers had almost identically sized groups as Kok and colleagues and produced significant results (Calvo-Merino et al., 2005; Yang, 2015). Additionally, in comparison to the study by Duty et al. (2012), the Kok et al. (2018) study identified the MNS in the precentral and parietal regions for each individual subject and did not look for neural activation differences outside of those areas. Thus, their results are not necessarily conflicting as Kok et al. (2018) did not assess differential activation in the cerebellum or visual cortex and Duty et al. (2012) did not report significant differences in the precentral or parietal regions during the observation tasks. It should also be noted that differences in cortical activation in the study by Duty et al. (2012) were much more apparent when subjects actually performed the laparoscopic task which could also explain the negative results in Kok et al. (2018). Studies investigating such active motor tasks are covered in the next section.

### Motor Task Performance (Various Brain Regions)

#### Prefrontal Cortex Activation

By far, the most common paradigm used to evaluate neural mechanisms of surgical expertise was motor task performance. Expert laparoscopic surgeons are known to have superior bimanual dexterity compared to junior surgeons and as decision-making becomes more intuitive, they can complete tasks at a faster rate. Leff et al. (2006) published the first study evaluating the use of functional near-infrared spectroscopy (fNIRS) to evaluate surgical expertise in a simple knot tying task. No differences in left PFC activation were found between three residents and four novices. This could be the result of a low sample size or due to the simplicity of the task in concert with the fact novices received training prior to and over the course of the study. The other study with results most similar to Leff et al. (2006) was published by Ohuchida et al. (2009). They investigated the effects of endoscopic surgery training on brain activation and found no difference in activation between experts and task naïve novices. In contrast to Leff et al., novices with exposure to training had increased activation in frontal channels. However, it is unclear where exactly the channels were positioned making comparisons across studies tenuous.

Most other studies have found differences between task naïve novices and intermediates or experts based on differential PFC activation. For example, Leff et al. (2007, 2008) published two studies using a group of 62 participants evaluating differences in brain activation of surgeons, residents, and medical students in a knot tying task. There was no difference in performance between surgeons and residents, but both groups outperformed medical students in terms of speed and efficiency. Similarly, there were no differences in neural activation between surgeons and residents, however, medical students demonstrated significant activation of the left PFC compared to the other groups and this activation diminished as the students became increasingly familiar with the task. Morris et al. (2015) also found that expert surgeons had decreased overall blood oxygen level-dependent (BOLD) signal changes upon fMRI in comparison to novices when tying knots by hand, whereas there was no difference in BOLD signal changes between expert surgeons and resident surgeons (Morris et al., 2015). Another study by Mylonas et al. (2012) corroborated these results in robotic surgery simulators using fNIRS. Compared to a group of 21 novices, a single expert showed significantly lower PFC activation in both free manipulation and Gaze-Contingent Motor Channeling (GCMC) paradigms of a visuomotor target localization task (Mylonas et al., 2012). This study was evidently limited by the sample size of the expert group since the primary purpose of the study was not to evaluate neural imaging differences between experts and novices, but rather to propose a novel method of providing haptic feedback based on visual information in robotic surgery training. These studies are some of many that demonstrate that surgical inexperience results in increased activation of the PFC and/or that many surgical tasks devised for these studies are insufficient to differentiate between the expertise level of residents versus attending surgeons.

However, there is one study in this review that found increased PFC activation in experts compared to medical students. James et al. (2011) assessed performance on a simulated natural orifice translumenal endoscopic surgery (NOTES) navigation task with simultaneous fNIRS (James et al., 2011). They found that experts significantly outperformed medical students on the task, but had greater lateral PFC activation. Importantly, when included in a random effects model, expertise was not found to be an independent predictor of PFC activation. The authors provide a number of potential reasons for the increased PFC activity in experts including that medical students were relying on luck and random movement rather than strategy for performing the task and that while the ‘experts’ were endoscopic experts they were not actually experts in NOTES procedures. These explanations are plausible and, importantly, similar neuroimaging results have not been seen since, although the NOTES task was not used in any other study in this review.

Notably, the use of questionable ‘experts’ was not limited to the James et al. (2011) study. Keles et al. (2021) performed expertise level classification using machine learning techniques. They compared PFC activity in novice medical students compared to attending surgeons in laparoscopic peg transfer and string threading tasks. No direct comparisons were made in terms of surgical task performance between groups (Keles et al., 2021). Applying basic machine learning techniques to the PFC activation data, they were able to classify subjects based on level of expertise with an accuracy of approximately 70–80%. However, some of the attending surgeons had as few as eight laparoscopic surgery experiences calling into question the actual expertise level of those subjects. An intriguing finding in this study was that while subjective evaluation of cognitive load positively correlated with PFC activation in novices, there was a negative correlation of the same variables in experts. The authors suggest this is due to the ‘expertise reversal effect’ that is purported to occur in situations where an experimental simulation is not optimally designed to replicate the real-world experience upon which the experts’ established motor skills were developed resulting in increased cognitive effort (Armougum et al., 2020). However, this effect was not noted in other experiments reviewed here despite likely suffering from similar difficulties replicating real experiences. Moreover, one would expect that if this were the case, experts would notice, and subjectively report, increased rather than decreased cognitive effort. Ensuring that ‘experts’ are indeed experts at the specific task used to evaluate surgical skill should be ensured in all future studies.

#### Motor Cortex, Supplementary Motor Area, and Other Regional Activations

In addition to differential PFC findings, some studies have reported on differences in motor cortex activity. One such study was conducted by Nemani et al. (2018). They used optical neuroimaging to investigate differences in bimanual motor skills using a laparoscopic pattern-cutting task (Nemani et al., 2018). They found that increased surgical expertise resulted in decreased prefrontal (PFC) activation and increased primary motor cortex (M1) and supplementary motor area (SMA) activation. These differences in activation were sufficient to accurately classify subjects by expertise level. Intriguingly, however, medical students who trained for 2 weeks performed as well or better than expert surgeons based on Fundamentals of Laparoscopic Surgery (FLS) performance scores. Along with the results of comparative performance on classification of surgical expertise level, this led the researchers to conclude that neuroimaging provides superior classification of surgical expertise than currently used surgical skill certification metrics such as FLS scores. A very similar result was reported by Shetty et al. (2016). Using a laparoscopic suturing task and fNIRS, they found an inverse correlation between PFC activation and expertise level (Shetty et al., 2016). Initially, experts outperformed residents and novices on the suturing task, however, after 2 weeks of training there were no performance differences between experts and novices. In this study, however, novices still had four to five times greater PFC activation than experts even when there was no discernable difference in performance metrics. This underscores the value of neuromonitoring for evaluation of surgical expertise and may reflect the difference in cognitive load between analytical versus intuitive task performance as defined in the Dreyfus model. However, it is also critical to notice that Nemani et al. (2018) provided comparisons of skilled novices to unskilled novices which showed that skilled novices did have decreased PFC activation compared to unskilled novices demonstrating that alterations to PFC activation were likely ongoing. Nemani et al. (2018) did not make direct comparisons between PFC activation of experts and skilled novices which would be necessary to fully corroborate the results of Shetty et al. (2016). Although the results of Shetty et al. (2016) and Nemani et al. (2018) are potentially compatible, in a feasibility study using fMRI to compare high-performing and low-performing medical students on laparoscopic tasks, Garbens et al. (2020) found no difference in brain activation between groups in an intracorporeal knot tying task despite high-performing medical students outperforming the low-performing medical students in terms of time taken and number of successful knots. This appears to contradict the findings of Nemani et al. (2018) however, the high-performing medical students in the Garbens et al. (2020) study had 1 h of training after going a full year without training and likely did not perform the suturing task at the level of an expert whereas the trained medical students in Nemani et al. (2018) had 2 weeks of training just prior to their imaging trials and did perform at the level of an expert.

Another study that found differences in regions outside the PFC was Duty et al. (2012). In addition to the motor observation tasks, they also had participants perform a laparoscopic peg transfer task and found increased PET response of the left insula and precentral gyrus as well as the right inferior occipital gyrus and precuneus in right-handed novices. These areas are associated with motor, visual spatial, and implicit learning. Experts, on the other hand, only activated the motor cortex to make the necessary movements and actually had decreased activation in the regions listed above where novices had increased activation corroborating the results of Nemani et al. (2018) and Duty et al. (2012). Furthermore, Duty et al. (2012) also noted that after minimal training, novices significantly improved performance of the task reaching an almost expert level performance after a practice period. Once again, like in Shetty et al. (2016), the differences in brain activation persisted despite similar performance on the task. The consistency of this finding across studies is reassuring and the implication is that although performance is similar, motor skill consolidation is still ongoing in novices. Unfortunately, Duty et al. (2012) did not perform expertise classification analyses to determine the accuracy of their imaging findings on correctly identifying experts.

#### Functional Connectivity

In addition to regional activation, increased connectivity between the frontal and motor regions has also previously been seen in early learners with inter-region connectivity decreasing over time with deliberate practice (Sun et al., 2007; Coynel et al., 2010). Andreu-Perez et al. (2016) hypothesized that expertise, resulting in neural plasticity, may be better evaluated through differences in this inter-region connectivity rather than regional activation, especially for the most difficult bimanual tasks that likely require more cognitive effort even in experts (Andreu-Perez et al., 2016). They found that novice surgeons had greater PFC-SMA, PFC-premotor cortex (PMC), and SMA-PMC connections during laparoscopic surgical tasks whereas SMA-M1 connections were similar, if not heightened, in experts. Moreover, they found that local connectivity within the frontal lobe was a superior classifier of expertise level compared to inter-region connectivity with the motor cortex. These results were consistent across three different tasks. Along similar lines, Nemani et al. (2021) investigated neural connectivity at various stages of expertise using fNIRS with wavelet coherence (WCO) and wavelet phase coherence (WPCO) metrics in both physical and virtual environments. WCO measures the correlation between two time series of neural activation. WPCO measures the variation in the difference of instantaneous phase between the two time series. The participants were eight surgeons, nine residents and 22 medical students, but comparisons were only made between surgeons versus residents and trained medical students versus untrained medical students (Nemani et al., 2021). In the physical simulator, WCO and WPCO metrics indicated greater connectivity between the central PFC (CPFC) and SMA in residents than in expert surgeons, however, this relationship was reversed in the virtual simulator. There were no differences in CPFC-SMA WCO metrics between untrained and trained medical students. There was, however, a significant difference in CPFC-SMA WPCO metrics with trained medical students having greater WPCO than untrained medical students. Although the results of the expert versus resident connectivity analyses in the physical simulator appear consistent with many prior studies, the implication of the most interesting results, namely the different results in the virtual simulator and the inconsistent results for WCO and WPCO in the analysis of medical students are unclear and were left unexplained. More detailed reports are required to determine the validity and relevance of these findings.

#### Whole Brain

In perhaps the best designed study for differentiating surgeons at the Competent, Proficient, or Expert levels of the Dreyfus framework, Guru et al. (2015) tested residents, fellows and attending surgeons in three tasks of varying complexity in robotic surgery while monitoring EEG metrics. Basic skills included ball placement tasks, suture pass task, and ring peg transfer tasks. The intermediate skill was the placement of a simple suture with knot tying. The advanced skill task was the performance of a simulated urethro-vesical anastomosis (UVA). Subjects were grouped based on the Dreyfus model [beginner vs. competent/proficient (C/P) vs. expert] in terms of robotic surgical skills based on responses to a questionnaire. In the basic skills, there were significant differences in performance in all metrics between beginners and C/Ps as well as between beginners and experts. The only performance difference between C/Ps and experts was in time taken with experts actually taking more time to complete the tasks. Beginners also had larger mental state and high-level engagement and overall cognitive score on EEG recording than competent/proficient participants and experts. C/Ps had greater high-level engagement, mental state, cognitive load and overall cognitive score than experts demonstrating once again that performance on tasks does not always correlate with proxies of motor task consolidation. Intriguingly, there were few task performance differences in the intermediate difficulty task. Specifically, beginners had significantly more arm-to-arm collisions than the other groups and appeared to take longer, but that difference did not reach statistical significance. It should be noted that there were only two participants in the beginner group, five in the C/P group, and three in the expert group. Once again, EEG showed that beginners had higher high-level engagement, mental states and overall cognitive scores than C/P subjects and experts. Cognitive load was also higher in beginners compared to experts. The same differences between beginners and experts were seen between C/P surgeons and experts. Beginners were not tested on the advanced task, but expert surgeons took about half the time it took C/Ps to complete the UVA. There were no other differences in task performance, however, there were significant differences in all EEG metrics with C/Ps surgeons having greater high-level engagement and higher scores on mental state, cognitive load and overall cognitive score. In terms of expertise level classification, Guru et al. (2015) found that high-level engagement and mental state metrics were the most accurate classifiers of surgical expertise. The consistency of expert surgeons requiring significantly less active cognition across a range of task difficulties again demonstrates the utility of neuromonitoring as a possible measure of surgical expertise that likely outperforms task performance metrics. Furthermore, future researchers should use tasks like the UVA task to help differentiate surgeons with significant training such as senior residents compared to attending surgeons. Whereas many of the tasks in other studies can be described as decomposed surgical tasks that even novices can quickly master, the UVA task is a more holistic task that can better differentiate between higher levels of decision making and awareness as described in the Dreyfus model. This is evident in the significant reduction in the time it took experts to complete the task versus C/Ps. The EEG metrics used in this study limit the specificity of the findings to any particular brain region. Although inferences could be made, using more targeted neuromonitoring methods in future studies employing the UVA task would be beneficial in determining the importance of specific brain regions and making comparisons across studies.

### Distraction (No Neuromonitoring)

Although most studies suggest that expert surgeons rely less on PFC to perform surgical tasks, Ghazanfar et al. (2015) noted that expert surgeons, like novices are significantly affected by distractions in difficult two-handed tasks suggesting PFC may still be significantly involved during more complex tasks and procedures. There were no studies with neuromonitoring that evaluated distraction in the context of surgical expertise, however, we discuss it briefly here as it is clearly an essential component of surgical expertise and should be tested in future neuromonitoring studies evaluating surgical expertise. A more comprehensive review on this topic was written by Mentis et al. (2016). The Dreyfus model of skill acquisition suggests experts should have more cognitive capacity available to handle distraction as their decision making is intuitive with minimal need for performance monitoring.

In surgical literature, distraction is best defined as a lapse in attention with a simultaneous orientation to a second task. This differentiates a distraction from an interruption which results in a lapse in task activity to attend to the second task (Healey et al., 2007; Mentis et al., 2016). In an electroencephalography (EEG) study, highly trained surgeons had decreased cognitive engagement while performing surgical procedures compared to less experienced surgeons (Guru et al., 2015). Thus it has been hypothesized that since experts may be less reliant on conscious processes during surgery, when the cognitive load of working memory increases with distraction tasks they should have greater processing capacity to manage both tasks simultaneously. Alternatively, expert surgeons may have enhanced cognitive efficiency rather than greater capacity and distinguishing between these two hypotheses presents a challenge for future research. Meneghetti et al. (2011) employed a dual-task distraction paradigm to test differences between expert and resident laparoscopic surgeons. The surgeons had to complete various laparoscopic procedures on virtual reality simulators while performing secondary tasks such as counting beeps or mental arithmetic. They had a low number of subjects, but demonstrated that the most difficult surgical task caused residents to have markedly worse performance on the secondary tasks compared to experts, who had stable error rates regardless of task combination. Further evidence of the differential effects of distraction based on level of expertise were shown in a study by Ghazanfar et al. (2015). They found that expert surgeons performed better on the primary task (surgery) than novices in distraction paradigms, but actually performed worse on the secondary task (distraction). The proposed implication is that expert surgeons are better able to identify and attend to the more pertinent task while tuning out less important stimuli. Moreover, there was a significant correlation between error probability, defined as errors per movement, and error rate, defined as errors per unit time, in novices, but not experts. Thus, it appeared that expert surgeons better identified high error probability situations and slowed down to ensure proper completion of the task whereas novices could not identify or did not adjust to the varying difficulty of procedural steps. Unfortunately, although neglect of secondary stimuli and identification of error prone parts of surgical procedures are intriguing possible markers of surgical expertise, their neural correlates have not yet been investigated. Possible brain regions that should be evaluated are the pre-SMA which has been specifically implicated in distraction resistant working memory and the anterior cingulate cortex which is associated with risk perception (Schmälzle et al., 2013; Wager et al., 2014). Although expert surgeons slowed down when faced with a procedural step with high error probability, it is essential to note that they also outperform junior colleagues in time restricted conditions, discussed in the next section.

### High-Temporal Demand (Prefrontal Cortex)

Most surgeries have significant risk of potentially deadly intraoperative complications, the mitigation of which often have critical time constraints. Manifestations of the difference in skill level between expert surgeons and their junior counterparts is often thought to be exacerbated when significant stress is imposed. This is known as the ‘choking effect’ and has been tested in high temporal demand paradigms.

Surgeons further along the Dreyfus model of expertise are less likely to fall victim to the choking effect as they generally take less time to perform tasks at baseline and their decision making is more intuitive, increasing the cognitive capacity available for task performance. Modi et al. (2018) demonstrated that the choking effect may be mediated by differential response in the PFC. Specifically, senior resident surgeons [post graduate year (PGY) 5] had increased activation in the PFC when required to complete laparoscopic suture in under 2 min, whereas novice surgeons (PGY 1–2) had PFC depression (Modi et al., 2018). Both groups had task deterioration and increased errors, but senior residents were able to maintain knot tensile strength suggesting they would be best categorized as competent surgeons under the Dreyfus framework. It would be valuable to conduct a similar study with attending surgeons to assess PFC activation and task deterioration in true experts. Using what appear to be the same subjects, Modi et al. (2019) performed a similar analysis stratifying subjects by the stability of their performance on the surgical task when under pressure. Using performance stability as their surrogate for expertise, they compared the top quartile to the bottom quartile and found that stable performance was correlated with increased ventrolateral and dorsolateral PFC engagement under temporal constraints. The subjects most susceptible to stress had a corresponding deactivation in those two areas.

The depression of PFC signal in novices when under time pressure has been previously noted in other domains (Al-Shargie et al., 2016). It has been demonstrated that PFC activity is modulated by hypothalamic-pituitary-adrenal axis activity in a dose dependent manner and that the capacity to increase lateral PFC activation may correlate with deliberate emotional regulation (Arnsten, 2009; Wheelock et al., 2016; Grabell et al., 2019). This suggests that overactivation of the HPA axis in novice surgeons may result in decreased attention to the task and increased risk of task failure. Although, in this study, there was no significant difference in any of the measured surgical performance outcomes to corroborate an increased risk of ‘choking.’ Modi et al. (2018) did find differences in heart rate between expert and junior surgeons (PGY 3-4) in high temporal demand conditions, but direct comparison of HPA activation through within-subject measurement of salivary cortisol or amylase in normal and high temporal demand conditions has not yet been made. Outside of the surgical realm, choking effects have been shown to have neural correlates in the ventral striatum, but potential neuroimaging biomarkers of surgical expertise in the ventral striatum have not been investigated (Chib et al., 2012).

There has also been increased interest in the neural correlates of positive versus negative emotional attractor coaching styles as well as self-criticism versus self-assurance during various learning tasks (Longe et al., 2010; Jack et al., 2013; Howard, 2015). Surgical specialties are now investigating the link between negative coaching styles and burnout, but the intersection of coaching style on choking effects has not yet been studied (Smith, 2020). However, novel research using neuroimaging has demonstrated other possible methods to protect against a choking effect in surgery. For example, a randomized control trial on the use of mindfulness tactics to reduce stress in residency demonstrated increased activation of the ventrolateral PFC in negative emotional states in surgical residents. Although not directly tested, it is possible that this intervention could protect against a choking effect, as these residents demonstrated enhanced executive function scores and motor skills than controls (Lebares et al., 2019). Additionally, it was shown that robotic surgery enhances PFC activation during instances of high temporal demand which was associated with better performance (Singh et al., 2018). Unfortunately, this study evaluated seven residents and one attending, but did not stratify subjects by level of expertise. The mechanism through which robotic surgery produces this effect is unclear, although robotic surgery has been previously associated with decreased cognitive load and stress response which may partially protect novice and intermediate surgeons against deficits in managing distraction and high temporal demand (van der Schatte Olivier et al., 2009; Stefanidis et al., 2010; Moore et al., 2015). Whether robotic surgery also improves performance in other task paradigms such as rapid intraoperative decision making, discussed next, has also not been studied.

### Decision-Making (Prefrontal Cortex)

An essential quality of an expert surgeon is the ability to make rapid and accurate intraoperative decisions. It has been shown that context specific cues enhance diagnostic accuracy for emergency medicine doctors (McRobert et al., 2013). Similarly, consistent with the Dreyfus model, it is thought that as surgeons gain experience they are likely better able to rely on context cues to generate intuitive, recognition-primed decisions whereas decision-making for surgeons with limited experience requires a more cognitively strenuous and analytical approach (Orasanu and Fischer, 1997; Flin et al., 2007; Leff et al., 2017).

Only one study has evaluated surgical decision making across expertise levels with neuromonitoring. Leff et al. (2017) performed a neuroimaging study of medical students, surgical residents, and surgical attending as they watched laparoscopic cholecystectomy videos and then deliberated over the appropriate next step (Leff et al., 2017). They found that medical students had significant activation of the dorsolateral PFC compared to both attending and residents in trials when the next step was not stated in the video. The dorsolateral PFC is known to have increased activation in the presence of significant uncertainty (Huettel et al., 2005; Leff et al., 2017). When the next step was stated in the video, none of the groups had activation of the PFC. These results suggest that, for residents and attending, the videos of the surgery provide sufficient context clues to generate a recognition-primed, intuitive decision whereas students must use the more cognitively intensive analytical approach unless they are primed by having the answer stated in the video. These preliminary results may indicate that the use of experiential and case study learning are superior to other techniques in developing decision-making capacity in students. Although these findings are also encouraging as a potential biomarker of surgical expertise, there is a significant difference in expertise between attending and residents that was not reflected by neuroimaging in this study. This is likely due to an inadequate difficulty level of the task.

## Conclusion

This review summarizes the current literature evaluating neural mechanisms of surgical expertise using neuromonitoring techniques and stratifying subjects across at least two levels of expertise.

**FIGURE 3:**
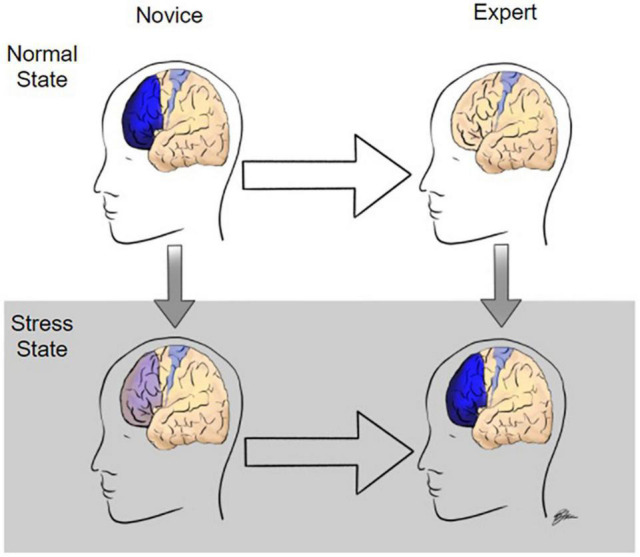
Modulation of prefrontal cortex activity during surgery as a function of expertise and stress level. In the normal state, when performing surgical tasks, novices have high levels of prefrontal cortex (PF) activation whereas experts activate only the motor cortex required to perform the task. Under stressful conditions, such as high temporal demand, experts have increased PFC activation whereas PFC activation is significantly diminished in novices.

The first goal of this review was to identify the strongest neural indicators of surgical expertise. Although the study of the neural correlates of surgical expertise is in its infancy, there is substantial evidence that differences in surgical expertise level can be delineated by differential activation of the PFC across multiple task modalities. Specifically, compared to novices, in motor task performance under normal state conditions, experts tend to have lower activation in the PFC and equivalent or increased activation in the motor cortex. The large majority of studies have used motor task performance paradigms to assess surgical expertise, however, similar PFC activation results have been seen in decision making tasks whereas high temporal demand tasks induce the opposite responses (Figure 3). Visualization and observation tasks, on the other hand, were found to produce differential activations in various regions including the visual cortex and cerebellum between novices and experts, although there have been very few studies in this realm limiting the validity of those results.

The second and third goals of this study was to identify the limitations of the current literature and suggest the most sensible future directions of study. Given the number of limitations, it seems most sensible to make a point of addressing them first. For example, a common limitation found in these studies was that the task difficulty was often insufficient to delineate between resident level and attending level surgical proficiency. In fact, novices could often attain attending level performance on these decomposed surgical tasks within a week of training. Since an impetus often cited for conducting these studies is providing a more objective measure of surgical skill in residency, surgical certification, and beyond, future studies of neural imaging biomarkers of surgical expertise should ensure their tasks require more advanced, holistic understanding and surgical skillset such as the UVA task in Guru et al. (2015).

Another common limitation was that many studies did not perform formal analyses of the ability of their neuromonitoring findings to accurately classify participants into their respective category of expertise. This type of analysis is essential in order to assess the actual utility of neuroimaging as a biomarker of expertise and/or surgical skill certification. It would also help in making comparisons of the quality of various biomarker signatures in studies that used different neuroimaging techniques or metrics. For example, it is currently difficult to compare the potential expertise classification value of findings in studies that quantified regional activation versus those that quantified functional connectivity.

Furthermore, it appears that attending level involvement was a limiting factor in multiple studies included in this review and may also have contributed to many studies being excluded from this review as there is a relatively large contingent of neuroimaging studies that only included surgical novices. Thus, increasing the involvement of attending in these research endeavors may improve study validity and enhance our ability to tease out differences between residents and attending. The low number of attending and occasional inclusion of questionable ‘experts’ combined with the simplicity of many of the tasks may also suggest the results reported in this review are more reflective of the neural correlates of surgical inexperience rather than surgical expertise.

Finally, most of the studies reviewed here used fNIRS as the neuroimaging modality. While it has many advantages in terms of feasibility and cost, one major disadvantage is that it provides no information about neural activity in non-cortical brain structures. By contrast, other modalities such as fMRI are not limited in this manner and could provide more utility for uncovering novel regions associated with surgical expertise in the future. Unfortunately, fMRI in particular has multiple well-known limitations including lack of portability, weaker temporal resolution, and lack of robustness to motion. All of these limitations are likely to be exacerbated as the difficulty of the surgical tasks increases as we suggested they should be. Therefore, substantial innovation and creativity is likely necessary to design an optimal experimental paradigm for understanding the neural mechanisms of surgical expertise in deeper brain structures.

There are also other considerations for future directions that are not directly linked to limitations highlighted in this review. For example, research into differences in neural activation and inter-region connectivity of the cerebellum and striatum may produce further important insights into the long-term encoding of surgical expertise. It will also be essential to fully elucidate whether surgical experts perform better in the presence of distraction tasks because they are absorbed in their primary task and neglect the secondary stimulus or because they have increased cognitive capacity. Based on existing literature, this is currently ambiguous. The mechanisms through which robotic surgery enhances the performance of junior surgeons across multiple domains also need to be clarified. Understanding the differences in neural activation during surgical decision-making tasks is paramount since this is one of the more evident hallmarks of surgical expertise. Future studies should also strive to incorporate advances in neural imaging technologies. Using artificial intelligence to analyze imaging data may produce novel and informative results. Future studies should investigate structural gray matter changes in addition to neuronal activation as studies in other fields have demonstrated transiently increased gray matter densities corresponding to various stages of motor training and cognitive load (Draganski et al., 2004; Driemeyer et al., 2008; Ceccarelli et al., 2009; Filippi et al., 2010; Gerber et al., 2014). Additionally, although currently unfeasible, the ability to demonstrate the necessity and sufficiency of various neural activations of surgical excellence may be possible in the more distant future with the creation of non-invasive, yet highly precise optogenetics (Chen et al., 2020). However, many challenges remain before this is remotely viable (Shen et al., 2020).

Although the evidence presented in this review is largely preliminary, residency directors should proactively consider the possibility of providing neural correlate feedback of surgical acumen in addition to common performance metrics to their pupils during their training (Ros et al., 2009). Similarly, future studies should strive to use longitudinal within-subjects design over the course of a residency or even a career. Although logistically arduous, this model would likely be most beneficial in demonstrating effects of increasing surgical expertise on regional brain activation and inter-region connectivity.

## Author Contributions

CK and DP conceptualized the project. TH wrote the original draft. TH, DT, RK, JB, DP, and CK edited, revised, and finalized the manuscript. RK illustrated Figure 3. CK supervised the project. All the authors contributed to the article and approved the submitted version.

## Conflict of Interest

The authors declare that the research was conducted in the absence of any commercial or financial relationships that could be construed as a potential conflict of interest.

## Publisher’s Note

All claims expressed in this article are solely those of the authors and do not necessarily represent those of their affiliated organizations, or those of the publisher, the editors and the reviewers. Any product that may be evaluated in this article, or claim that may be made by its manufacturer, is not guaranteed or endorsed by the publisher.

## References

[B1] Alderson D. (2010). Developing expertise in surgery. *Med. Teach.* 32 830–836. 20854159 10.3109/01421591003695329

[B2] Al-Shargie F. Kiguchi M. Badruddin N. Dass S. C. Hani A. F. Tang T. B. (2016). Mental stress assessment using simultaneous measurement of EEG and fNIRS. *Biomed. Opt. Express* 7 3882–3898. 10.1364/BOE.7.003882 27867700 PMC5102531

[B3] Andreu-Perez J. Leff D. R. Shetty K. Darzi A. Yang G.-Z. (2016). Disparity in frontal lobe connectivity on a complex bimanual motor task aids in classification of operator skill level. *Brain Connect.* 6 375–388. 10.1089/brain.2015.0350 26899241

[B4] Armougum A. Gaston-Bellegarde A. Marle C. J.-L. Piolino P. (2020). Expertise reversal effect: cost of generating new schemas. *Comput. Hum. Behav.* 111:106406.

[B5] Arnsten A. F. (2009). Stress signalling pathways that impair prefrontal cortex structure and function. *Nat. Rev. Neurosci.* 10 410–422. 10.1038/nrn2648 19455173 PMC2907136

[B6] Calvo-Merino B. Glaser D. E. Grèzes J. Passingham R. E. Haggard P. (2005). Action observation and acquired motor skills: an fMRI study with expert dancers. *Cereb. Cortex* 15 1243–1249. 10.1093/cercor/bhi007 15616133

[B7] Ceccarelli A. Rocca M. A. Pagani E. Falini A. Comi G. Filippi M. (2009). Cognitive learning is associated with gray matter changes in healthy human individuals: a tensor-based morphometry study. *NeuroImage* 48 585–589. 10.1016/j.neuroimage.2009.07.009 19615452

[B8] Chen R. Gore F. Nguyen Q.-A. Ramakrishnan C. Patel S. Kim S. H. (2020). Deep brain optogenetics without intracranial surgery. *Nat. Biotech.* 39 161–164. 10.1038/s41587-020-0679-9 33020604 PMC7878426

[B9] Chib V. S. De Martino B. Shimojo S. O’Doherty J. P. (2012). Neural mechanisms underlying paradoxical performance for monetary incentives are driven by loss aversion. *Neuron* 74 582–594. 10.1016/j.neuron.2012.02.038 22578508 PMC3437564

[B10] Coynel D. Marrelec G. Perlbarg V. Pélégrini-Issac M. Van de Moortele P.-F. Ugurbil K. (2010). Dynamics of motor-related functional integration during motor sequence learning. *Neuroimage* 49 759–766. 10.1016/j.neuroimage.2009.08.048 19716894 PMC2764831

[B11] Draganski B. Gaser C. Busch V. Schuierer G. Bogdahn U. May A. (2004). Changes in grey matter induced by training. *Nature* 427 311–312. 14737157 10.1038/427311a

[B12] Dreyfus H. Zsambok C. Klein G. J. M. (1997). *Naturalistic Decision Making.* Hove: Psychology Press.

[B13] Dreyfus S. Dreyfus H. (1980). *A Five-Stage Model of the Mental Activities Involved in Directed Skill Acquisition.* Berkeley: University of California.

[B14] Driemeyer J. Boyke J. Gaser C. Büchel C. May A. (2008). Changes in gray matter induced by learning—revisited. *PLoS One* 3:e2669. 10.1371/journal.pone.0002669 18648501 PMC2447176

[B15] Duty B. Andonian S. Ma Y. Peng S. Shapiro E. Dhawan V. (2012). Correlation of laparoscopic experience with differential functional brain activation: a positron emission tomography study with oxygen 15–labeled water. *Arch. Surg*. 147 627–632. 10.1001/archsurg.2012.807 22802056

[B16] Fahy A. S. Jamal L. Carrillo B. Gerstle J. T. Nasr A. Azzie G. (2019). Refining how we define laparoscopic expertise. *J. Laparoendosc. Adv. Surg. Tech*. 29 396–401. 10.1089/lap.2018.0254 30650004

[B17] Farrow D. Reid M. (2012). The contribution of situational probability information to anticipatory skill. *J. Sci. Med. Sport* 15 368–373. 10.1016/j.jsams.2011.12.007 22406067

[B18] Filippi M. Ceccarelli A. Pagani E. Gatti R. Rossi A. Stefanelli L. (2010). Motor learning in healthy humans is associated to gray matter changes: a tensor-based morphometry study. *PLoS One* 5:e10198. 10.1371/journal.pone.0010198 20419166 PMC2855363

[B19] Flin R. Youngson G. Yule S. (2007). How do surgeons make intraoperative decisions?. *Qual. Saf. Health Care* 16 235–239. 17545353 10.1136/qshc.2006.020743 PMC2464983

[B20] Gabrieli J. D. E. (1998). Cognitive neuroscience of human memory. *Ann. Rev. Psychol.* 49 87–115. 9496622 10.1146/annurev.psych.49.1.87

[B21] Garbens A. Armstrong B. A. Louridas M. Tam F. Detsky A. S. Schweizer T. A. (2020). Brain activation during laparoscopic tasks in high- and low-performing medical students: a pilot fMRI study. *Surg. Endosc.* 34 4837–4845. 10.1007/s00464-019-07260-5 31754848

[B22] Gerber P. Schlaffke L. Heba S. Greenlee M. W. Schultz T. Schmidt-Wilcke T. (2014). Juggling revisited — a voxel–based morphometry study with expert jugglers. *Neuroimage* 95 320–325. 10.1016/j.neuroimage.2014.04.023 24736178

[B23] Ghazanfar M. A. Cook M. Tang B. Tait I. Alijani A. (2015). The effect of divided attention on novices and experts in laparoscopic task performance. *Surg. Endosc*. 29 614–619. 10.1007/s00464-014-3708-2 25030475

[B24] Gilboa A. Marlatte H. (2017). Neurobiology of schemas and schema-mediated memory. *Trends Cogn. Sci.* 21 618–631. 10.1016/j.tics.2017.04.013 28551107

[B25] Grabell A. S. Huppert T. J. Fishburn F. A. Li Y. Hlutkowsky C. O. Jones H. M. (2019). Neural correlates of early deliberate emotion regulation: young children’s responses to interpersonal scaffolding. *Dev. Cogn. Neurosci*. 40:100708. 10.1016/j.dcn.2019.100708 31577981 PMC6974895

[B26] Guo D. Yang J. (2020). Interplay of the long axis of the hippocampus and ventromedial prefrontal cortex in schema-related memory retrieval. *Hippocampus* 30 263–277. 10.1002/hipo.23154 31490611

[B27] Guru K. A. Esfahani E. T. Raza S. J. Bhat R. Wang K. Hammond Y. (2015). Cognitive skills assessment during robot-assisted surgery: separating the wheat from the chaff. *BJU Int.* 115 166–174. 10.1111/bju.12657 24467726

[B28] Healey A. N. Primus C. P. Koutantji M. (2007). Quantifying distraction and interruption in urological surgery. *Qual. Saf. Health Care* 16 135–139. 10.1136/qshc.2006.019711 17403761 PMC2653151

[B29] Hoffman R. (1998). *Exploring Expertise.* Basingstoke: Macmillan.

[B30] Howard A. R. (2015). Coaching to vision versus coaching to improvement needs: a preliminary investigation on the differential impacts of fostering positive and negative emotion during real time executive coaching sessions. *Front. Psychol*. 6:455. 10.3389/fpsyg.2015.00455 25964768 PMC4408757

[B31] Howell W. S. (1986). *The Empathic Communicator.* Long Grove: Waveland Press.

[B32] Huettel S. A. Song A. W. McCarthy G. (2005). Decisions under uncertainty: probabilistic context influences activation of prefrontal and parietal cortices. *J. Neurosci.* 25 3304–3311. 10.1523/JNEUROSCI.5070-04.2005 15800185 PMC6724903

[B33] Jack A. I. Boyatzis R. E. Khawaja M. S. Passarelli A. M. Leckie R. L. (2013). Visioning in the brain: an fMRI study of inspirational coaching and mentoring. *Soc. Neurosci.* 8 369–384. 10.1080/17470919.2013.808259 23802125

[B34] James D. R. C. Orihuela-Espina F. Leff D. R. Sodergren M. H. Athanasiou T. Darzi A. W. (2011). The ergonomics of natural orifice translumenal endoscopic surgery (notes) navigation in terms of performance, stress, and cognitive behavior. *Surgery* 149 525–533. 10.1016/j.surg.2010.11.019 21295807

[B35] Kavic M. S. (2012). Teaching and learning of surgery. *JSLS* 16 341–344. 23318057 10.4293/108680812X13427982376103 PMC3535801

[B36] Keles H. O. Cengiz C. Demiral I. Ozmen M. M. Omurtag A. (2021). High density optical neuroimaging predicts surgeons’s subjective experience and skill levels. *PLoS One* 16:e0247117. 10.1371/journal.pone.0247117 33600502 PMC7891714

[B37] Kok E. De Bruin A. B. van Geel K. Gegenfurtner A. Heyligers I. Sorger B. (2018). The neural implementation of surgical expertise within the mirror-neuron system: an fMRI study. *Front. Hum. Neurosci.* 12:291. 10.3389/fnhum.2018.00291 30079016 PMC6062624

[B38] Koziol L. F. Budding D. Andreasen N. D’Arrigo S. Bulgheroni S. Imamizu H. (2014). Consensus paper: the cerebellum’s role in movement and cognition. *Cerebellum* 13 151–177. 10.1007/s12311-013-0511-x 23996631 PMC4089997

[B39] Lebares C. C. Guvva E. V. Olaru M. Sugrue L. P. Staffaroni A. M. Delucchi K. L. (2019). Efficacy of mindfulness-based cognitive training in surgery: additional analysis of the mindful surgeon pilot randomized clinical trial. *JAMA Netw. Open* 2:e194108. 10.1001/jamanetworkopen.2019.4108 31125095 PMC6632137

[B40] Leff D. Koh P. H. Aggarwal R. Leong J. Deligianni F. Elwell C. (2006). “Optical mapping of the frontal cortex during a surgical knot-tying task, a feasibility study,” in *Paper presented at: Medical Imaging and Augmented Reality*, eds Yang G. Z. Jiang T. Shen D. Gu L. Yang J. (Berlin: Springer).

[B41] Leff D. R. Elwell C. E. Orihuela-Espina F. Atallah L. Delpy D. T. Darzi A. W. (2008). Changes in prefrontal cortical behaviour depend upon familiarity on a bimanual co-ordination task: an fNIRS study. *Neuroimage* 39 805–813. 10.1016/j.neuroimage.2007.09.032 17964187

[B42] Leff D. R. Orihuela-Espina F. Atallah L. Darzi A. Yang G. Z. (2007). Functional near infrared spectroscopy in novice and expert surgeons–a manifold embedding approach. *Med. Image Comput. Comput. Assist. Interv.* 10 270–277. 10.1007/978-3-540-75759-7_33 18044578

[B43] Leff D. R. Yongue G. Vlaev I. Orihuela-Espina F. James D. Taylor M. J. (2017). “Contemplating the next maneuver”: functional neuroimaging reveals intraoperative decision-making strategies. *Ann. Surg.* 265 320–330. 10.1097/SLA.0000000000001651 28059960

[B44] Leppink J. (2017). Cognitive load theory: practical implications and an important challenge. *J. Taibah Univ. Med. Sci.* 12 385–391. 10.1016/j.jtumed.2017.05.003 31435268 PMC6694886

[B45] Longe O. Maratos F. A. Gilbert P. Evans G. Volker F. Rockliff H. (2010). Having a word with yourself: neural correlates of self-criticism and self-reassurance. *Neuroimage* 49 1849–1856. 10.1016/j.neuroimage.2009.09.019 19770047

[B46] Lum J. A. G. Conti-Ramsden G. Page D. Ullman M. T. (2012). Working, declarative and procedural memory in specific language impairment. *Cortex* 48 1138–1154. 10.1016/j.cortex.2011.06.001 21774923 PMC3664921

[B47] Macnamara B. N. Maitra M. (1993). The role of deliberate practice in expert performance: revisiting Ericsson, Krampe & Tesch-Römer. *R. Soc. Open Sci.* 6:190327. 10.1098/rsos.190327 31598236 PMC6731745

[B48] McRobert A. P. Causer J. Vassiliadis J. Watterson L. Kwan J. Williams M. A. (2013). Contextual information influences diagnosis accuracy and decision making in simulated emergency medicine emergencies. *BMJ Qual. Saf*. 22 478–484. 10.1136/bmjqs-2012-000972 23396852

[B49] Meneghetti A. T. Pachev G. Zheng B. Panton O. N. Qayumi K. (2011). Objective assessment of laparoscopic skills: dual-task approach. *Surg. Innov.* 19 452–459. 10.1177/1553350611430673 22170894

[B50] Mentis H. M. Chellali A. Manser K. Cao C. G. L. Schwaitzberg S. D. (2016). A systematic review of the effect of distraction on surgeon performance: directions for operating room policy and surgical training. *Surg. Endosc*. 30 1713–1724. 10.1007/s00464-015-4443-z 26194261 PMC5663645

[B51] Modi H. N. Singh H. Fiorentino F. Orihuela-Espina F. Athanasiou T. Yang G.-Z. (2019). Association of residents’ neural signatures with stress resilience during surgery. *JAMA Surg.* 154:e192552. 10.1001/jamasurg.2019.2552 31389994 PMC6686757

[B52] Modi H. N. Singh H. Orihuela-Espina F. Athanasiou T. Fiorentino F. Yang G.-Z. (2018). Temporal stress in the operating room: brain engagement promotes “coping” and disengagement prompts “choking”. *Ann. Surg.* 267 683–691. 10.1097/SLA.0000000000002289 28489681

[B53] Modi H. N. Singh H. Yang G.-Z. Darzi A. Leff D. R. (2017). A decade of imaging surgeons’ brain function (part ii): a systematic review of applications for technical and nontechnical skills assessment. *Surgery* 162 1130–1139. 10.1016/j.surg.2017.09.002 29079277

[B54] Moore L. J. Wilson M. R. McGrath J. S. Waine E. Masters R. S. Vine S. J. (2015). Surgeons’ display reduced mental effort and workload while performing robotically assisted surgical tasks, when compared to conventional laparoscopy. *Surg. Endosc*. 29 2553–2560. 10.1007/s00464-014-3967-y 25427414

[B55] Morris M. C. Frodl T. D’Souza A. Fagan A. J. Ridgway P. F. (2015). Assessment of competence in surgical skills using functional magnetic resonance imaging: a feasibility study. *J. Surg. Educ.* 72 198–204. 10.1016/j.jsurg.2014.09.007 25439177

[B56] Mylonas G. P. Kwok K.-W. James D. R. C. Leff D. Orihuela-Espina F. Darzi A. (2012). Gaze-contingent motor channelling, haptic constraints and associated cognitive demand for robotic mis. *Med. Image Anal.* 16 612–631. 10.1016/j.media.2010.07.007 20889367

[B57] Nemani A. Kamat A. Gao Y. Yucel M. Gee D. Cooper C. (2021). Functional brain connectivity related to surgical skill dexterity in physical and virtual simulation environments. *Neurophotonics* 8:015008. 10.1117/1.NPh.8.1.015008 PMC7927423 33681406

[B58] Nemani A. Yücel M. A. Kruger U. Gee D. W. Cooper C. Schwaitzberg S. D. (2018). Assessing bimanual motor skills with optical neuroimaging. *Sci. Adv.* 4:eaat3807. 10.1126/sciadv.aat3807 30306130 PMC6170034

[B59] Nicoll R. A. (2017). A brief history of long-term potentiation. *Neuron* 93 281–290. 10.1016/j.neuron.2016.12.015 28103477

[B60] Ohuchida K. Kenmotsu H. Yamamoto A. Sawada K. Hayami T. Morooka K. (2009). The frontal cortex is activated during learning of endoscopic procedures. *Surg. Endosc.* 23 2296–2301. 10.1007/s00464-008-0316-z 19172351

[B61] Orasanu J. Fischer U. (1997). *Finding Decisions in Natural Environments: The View from the Cockpit.* Mahwah: Lawrence Erlbaum Associates, 343–357.

[B62] Rajmohan V. Mohandas E. (2007). Mirror neuron system. *Indian J. Psychiatry* 49 66–69. 20640069 10.4103/0019-5545.31522 PMC2900004

[B63] Rizzolatti G. Craighero L. (2004). The mirror-neuron system. *Ann. Rev. Neurosci.* 27 169–192. 15217330 10.1146/annurev.neuro.27.070203.144230

[B64] Roca A. Williams A. M. (2016). Expertise and the interaction between different perceptual-cognitive skills: implications for testing and training. *Front. Psychol*. 7:792. 10.3389/fpsyg.2016.00792 27252677 PMC4879788

[B65] Ros T. Moseley M. J. Bloom P. A. Benjamin L. Parkinson L. A. Gruzelier J. H. (2009). Optimizing microsurgical skills with EEG neurofeedback. *BMC Neurosci.* 10:87. 10.1186/1471-2202-10-87 19630948 PMC2723116

[B66] Sánchez A. Rodríguez O. Sánchez R. Benítez G. Pena R. Salamo O. (2014). Laparoscopic surgery skills evaluation: analysis based on accelerometers. *JSLS* 18:e2014.00234. 10.4293/JSLS.2014.00234 25489218 PMC4254482

[B67] Savelsbergh G. J. P. Van der Kamp J. Williams A. M. Ward P. (2005). Anticipation and visual search behaviour in expert soccer goalkeepers. *Ergonomics* 48 1686–1697. 16338733 10.1080/00140130500101346

[B68] Schmälzle R. Häcker F. Renner B. Honey C. J. Schupp H. T. (2013). Neural correlates of risk perception during real-life risk communication. *J. Neurosci.* 33 10340–10347. 10.1523/JNEUROSCI.5323-12.2013 23785147 PMC3755178

[B69] Shen Y. Campbell R. E. Côté D. C. Paquet M. E. (2020). Challenges for therapeutic applications of opsin-based optogenetic tools in humans. *Front. Neural Circuits* 14:41. 10.3389/fncir.2020.00041 32760252 PMC7373823

[B70] Shetty K. Leff D. R. Orihuela-Espina F. Yang G.-Z. Darzi A. (2016). Persistent prefrontal engagement despite improvements in laparoscopic technical skill. *JAMA Surg.* 151 682–684. 10.1001/jamasurg.2016.0050 27028901

[B71] Singh H. Modi H. N. Ranjan S. Dilley J. W. R. Airantzis D. Yang G.-Z. (2018). Robotic surgery improves technical performance and enhances prefrontal activation during high temporal demand. *Ann. Biomed. Eng*. 46 1621–1636. 10.1007/s10439-018-2049-z 29869104 PMC6153983

[B72] Smith J. M. (2020). Surgeon coaching: why and how. *J. Pediatr. Orthop.* 40 S33–S37. 10.1097/BPO.0000000000001541 32502069

[B73] Spalding K. N. Jones S. H. Duff M. C. Tranel D. Warren D. E. (2015). Investigating the neural correlates of schemas: ventromedial prefrontal cortex is necessary for normal schematic influence on memory. *J. Neurosci.* 35 15746–15751. 10.1523/JNEUROSCI.2767-15.2015 26609165 PMC4659831

[B74] Stefanidis D. Wang F. Korndorffer J. R. Jr. Dunne J. B. Scott D. J. (2010). Robotic assistance improves intracorporeal suturing performance and safety in the operating room while decreasing operator workload. *Surg. Endosc*. 24 377–382. 10.1007/s00464-009-0578-0 19536599

[B75] Sun F. T. Miller L. M. Rao A. A. D’Esposito M. (2007). Functional connectivity of cortical networks involved in bimanual motor sequence learning. *Cereb. Cortex* 17 1227–1234. 10.1093/cercor/bhl033 16855008

[B76] van der Schatte Olivier R. H. Van’t Hullenaar C. D. Ruurda J. P. Broeders I. A. (2009). Ergonomics, user comfort, and performance in standard and robot-assisted laparoscopic surgery. *Surg. Endosc*. 23 1365–1371. 10.1007/s00464-008-0184-6 18855053 PMC2687080

[B77] van Kesteren M. T. R. Ruiter D. J. Fernández G. Henson R. N. (2012). How schema and novelty augment memory formation. *Trends Neurosci.* 35 211–219. 10.1016/j.tins.2012.02.001 22398180

[B78] Wager T. D. Spicer J. Insler R. Smith E. E. (2014). The neural bases of distracter-resistant working memory. *Cogn. Affect. Behav. Neurosci.* 14 90–105. 10.3758/s13415-013-0226-y 24366656 PMC3972280

[B79] Wang Z. Majewicz Fey A. (2018). Deep learning with convolutional neural network for objective skill evaluation in robot-assisted surgery. *Int. J. Comput. Assist. Radiol. Surg*. 13 1959–1970. 10.1007/s11548-018-1860-1 30255463

[B80] Wheelock M. D. Harnett N. G. Wood K. H. Orem T. R. Granger D. A. Mrug S. (2016). Prefrontal cortex activity is associated with biobehavioral components of the stress response. *Front. Hum. Neurosci*. 10:583. 10.3389/fnhum.2016.00583 27909404 PMC5112266

[B81] Willams A. M. Hodges N. J. North J. S. Barton G. (2006). Perceiving patterns of play in dynamic sport tasks: investigating the essential information underlying skilled performance. *Perception* 35 317–332. 10.1068/p5310 16619949

[B82] Yang J. (2015). The influence of motor expertise on the brain activity of motor task performance: a meta-analysis of functional magnetic resonance imaging studies. *Cogn. Affect. Behav. Neurosci*. 15 381–394. 10.3758/s13415-014-0329-0 25450866

[B83] Young J. Q. Van Merrienboer J. Durning S. Ten Cate O. (2014). Cognitive load theory: implications for medical education: AMEE guide no. 86. *Med. Teach.* 36 371–384. 10.3109/0142159X.2014.889290 24593808

[B84] Zhu Y. Wang R. Wang Y. (2016). A comparative study of the impact of theta-burst and high-frequency stimulation on memory performance. *Front. Hum. Neurosci*. 10:19. 10.3389/fnhum.2016.00019 26869903 PMC4737909

